# Longitudinally Extensive Transverse Myelitis with Intramedullary Metastasis of Small-Cell Lung Carcinoma: An Autopsy Case Report

**DOI:** 10.1155/2013/305670

**Published:** 2013-12-22

**Authors:** Kenya Nishioka, Ryota Tanaka, Satoshi Tsutsumi, Hideki Shimura, Yutaka Oji, Harumi Saeki, Yukimasa Yasumoto, Masanori Ito, Nobutaka Hattori, Takao Urabe

**Affiliations:** ^1^Department of Neurology, Juntendo University School of Medicine, 2-1-1 Hongo, Bunkyo, Tokyo, Japan; ^2^Department of Neurosurgery, Juntendo University Urayasu Hospital, 2-1-1 Tomioka, Urayasu, Chiba, Japan; ^3^Department of Neurology, Juntendo University Urayasu Hospital, Chiba, Japan; ^4^Department of Pathology, Juntendo University Urayasu Hospital, 2-1-1 Tomioka, Urayasu, Chiba, Japan

## Abstract

*Background*. Longitudinally extensive transverse myelitis (LETM) is characterized by spinal cord inflammation extending vertically through three or more vertebral segments. The widespread use of MRI revealed LETM more frequency than ever. We report the case of a patient with pathologically confirmed small-cell lung carcinoma metastasis into the spinal cord presenting as LETM. *Case Presentation*. A 74-year-old man developed rapidly progressive sensorimotor disturbance and vesicorectal dysfunction. T2-weighted magnetic resonance imaging of the spine revealed LETM at the level of from T3 to conus medullaris; gadolinium enhancement showed concurrent tumor in the thoracic spinal cord from T10 to T11. Systemic survey identified a nodular mass in the lung that was verified as small-cell carcinoma. Following initial failed treatment by high-dose steroid, the patient underwent an emergent microsurgical tumor resection. Histological examination was identical with the lung carcinoma. The patient died of tumor progression at the 47th day after admission. At autopsy, only changes of edema were found in the gray matter of the cord, while tumor cells were not noted in it. *Conclusion*. Metastasis may rarely present symptoms of LETM. Prompt identification of underlying etiology by contrast examination and systemic survey is crucial for the patient assumed as LETM.

## 1. Introduction

Longitudinally extensive transverse myelitis (LETM) is characterized by spinal cord inflammation extending vertically through three or more vertebral segments [1]. The widespread use of magnetic resonance imaging (MRI) has revealed small cell lung cancer (SCLC) to be associated with many primary pathologies, including inflammatory diseases (collagen disease, neuromyelitis optica, Sjögren's syndrome, systemic lupus erythematosus, neuro-Behçet's disease, and sarcoidosis); infectious diseases (human T-lymphotropic virus-1-associated myelopathy, human immunodeficiency virus infection, and adverse reaction to influenza vaccination); and noninflammatory diseases (intramedullary spinal neoplasms, spinal cord infarction, and spinal dural arteriovenous fistula) [1–14]. LETM has a heterogeneous pathogenesis, but reported cases associated with malignant disorders were very rare (Table 1). Here, we report a case of LETM due to intramedullary metastasis of small-cell lung carcinoma (SCLC), with rapid progression of paraplegia and sensory disturbance of the lower limbs. This patient presented with LETM on whole-spine MRI; despite intensive treatment, he died 47 days after admission. An autopsy was performed to assess the pathological findings of LETM.

## 2. Case Presentation

**Figure 1:**
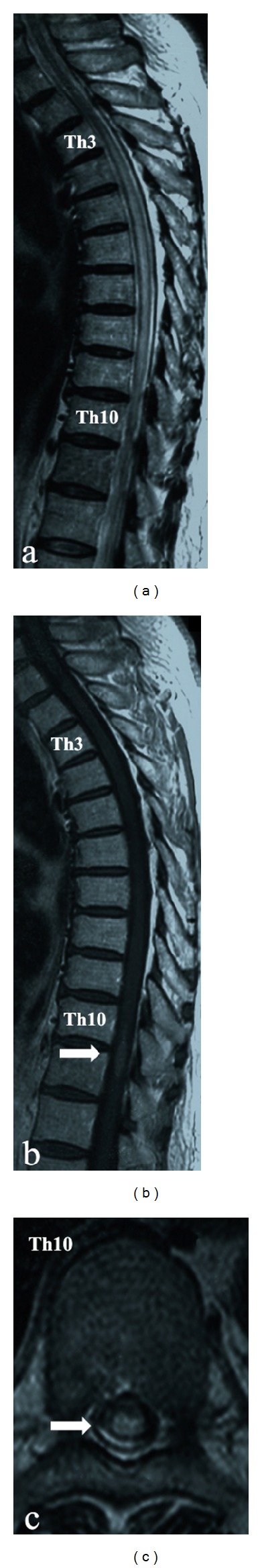
Thoracic magnetic resonance imaging reveals an intramedullary long spinal cord lesion from T3 to lower levels at 1 day after admission. (a) Sagittal T2-weighted image. (b) Gadolinium enhancement of the lesion at T10-T11. (c) Axial T2-weighted image at T10, revealing a high-intensity signal in the center of the spinal cord.

**Figure 2:**
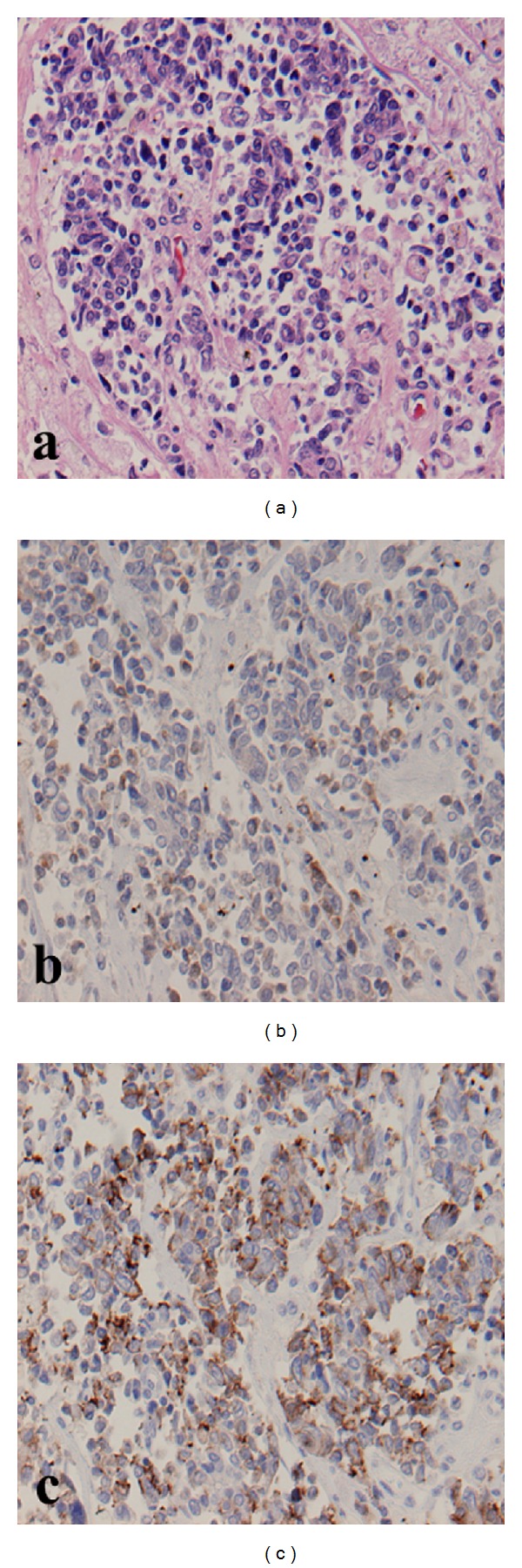
(a–c) Histopathology of the surgically resected spinal cord tumor, revealing a high nuclear/cytoplasmic ratio with necrosis. The sections were stained by hematoxylin and eosin (H&E) (a) following immunostaining with synaptophysin (b) and CD56 antibodies (c). Both synaptophysin- and CD56-positive cells were observed. The tumor was attributed to small-cell lung carcinoma metastasis. Original magnification ×100.

A 74-year-old man, a habitual smoker, developed gait disturbance with exacerbation in the following 8 days. At the time of admission, he had difficulty in walking without assistance. His past medical history was unremarkable. On admission, he was fully alert. Neurological examination revealed sensory deficits, predominantly of deep sensation, and sphincter dysfunction with constipation and urinary disturbance. Manual muscle tests of all limbs indicated no paralysis, but lower limbs exhibited mild spasticity. Patellar and Achilles tendon reflexes were bilaterally brisk. Vibratory sensation was bilaterally diminished below the anterior superior iliac spine, but temperature and pain modalities were preserved. All tested hematological and biochemical parameters were within normal, including thyroid function, serum immunoglobulin, and serum vitamin B12. The levels of anti-SSA and anti-SSB antibodies were below 7.0 U/mL. Progastrin-releasing peptide level was elevated to 189 pg/mL (normal < 70 pg/mL), and carcinoembryonic antigen level was also considerably high (59.7 ng/mL, normal < 5.0 ng/mL). MRI revealed extensive intramedullary abnormal intensity from the upper thoracic cord down to conus medullaris. An intramedullary spinal cord tumor was detected at T10-T11 that was enhanced by gadolinium (Figure 1). Chest computed tomography (CT) scans revealed a small nodular lesion of 18 × 21 mm with spicula in the right lung S1 and hilar lymph node swelling on the right side, verified as small-cell carcinoma from biopsy specimen. Abdominal CT scans with iopamidol did not detect metastasis to the liver, spleen, or kidney. The metastasis led to gait disturbance and other sensorimotor symptoms. Initially, the patient was treated with intravenous methylprednisolone at 1,000 mg/day for 3 days, followed by oral prednisolone at 50 mg/day, which are not effective. Then he underwent an emergent microsurgical tumor resection at 9 days after admission. The mass obtained from surgical resection contained atypical cells with chromatin condensation that were synaptophysin/CD56 positive but chromogranin A negative (Figures 2(a)−2(c)). After the resection, lumbar MRI indicated improvement of LETM. Neurological functions continued to deteriorate, and the patient died of pulmonary hemorrhage 47 days after admission. Autopsy confirmed a 20 mm SCLC in the right upper lung with right hilar adenopathy. Spinal sections showed mild edematous changes associated with the infiltration of lymphocytes and plasma cells in the gray matter. However, there were no cancer cells in the spinal cord, even at the edges of sections from T10-L1. Histological examination of the whole brain revealed modest lymphocyte proliferation in the cortex and around the lateral ventricles but no sign of metastasis.

In contrast to LETM revealed by MRI and biopsy results indicating SCLC metastasis into the spine, postmortem pathology revealed only mild edematous changes localized around the dorsal horn. Furthermore, there were no signs of gray matter tumors or meningeal carcinomatosis and no pencil-shaped softening indicative of necrosis in autopsy spine samples.

## 3. Discussion

In our patient, metastasis of lung carcinoma to the spinal cord resulted in intramedullary spinal cord tumors and LETM. These tumors are observed in only 1%-2% patients with cancer at autopsy and in only 8.5% cases with central nervous system metastasis, most commonly from lung carcinoma [15]. LETM is detected on MRI more frequently than ever before. However, the precise mechanism underlying LETM development remains unknown. To our knowledge, It is very rare that LETM is caused by metastasis.

The pathological findings in the spinal cord of our patient indicate mild changes of edema without malignant cell invasion or metastasis. These findings suggest that LETMs themselves are reversible by ameliorating edema or temporary inflammation. Ohnaka et al. described that LETM was the result of lung carcinoma that completely disappeared after tumor resection [3]. They concluded that edema was the main pathology associated with LETM. In fact, many previous reports prove that LETMs due to inflammatory diseases can be ameliorated by medications (Table 1). Even intramedullary spinal cord metastases can be temporarily treated by tumor resection [3]. We speculate that gray matter is more sensitive to damage by venous congestion than white matter because of the difference in water distribution. This difference can lead to intramedullary LETMs. There were some discrepancies between clinical manifestations and spinal MRI findings in the case described here. These particular findings also confirm the slight differences in LETM.

Our patient developed paraplegia as the initial symptom. Intramedullary spinal cord metastases have a high frequency (20%–40%) of neurological deficits as the first symptom before a diagnosis of cancer [15]. Kalayci et al. reported an average 52 days from neurological symptom onset to cancer diagnosis. Because this gap may result in cancer exacerbation, LETM should be considered as a critical sign of metastasis or primary tumors.

LETM can be caused by diverse spectrum of etiology; however, most of the patients presented in association with autoimmune disorders. (Table 1). We explored papers describing LETM appearing on MRI in PubMed in the past 3 years (Table 1). Many reports described discrepancies between the findings of MRI and clinical symptoms. Their initial symptoms were limited weakness and numbness in lower limbs, despite the broad emergence of LETM [2–14]. Despite mild changes in LETM, the prognosis in cases with metastasis is generally poor, with a 3-4-month mortality rate of approximately 80% [15]. These results depend on the stage of malignancy. Metastasis itself is associated with poor prognosis.

## 4. Conclusions

LETM may present as symptoms of intramedullary spinal cord metastasis. Cases complicated with primary malignant disorders are heralded by poor prognosis, but some may result in a significant symptom relief by surgical resection. We emphasize that precise and rapid diagnosis of LETM is needed to optimize treatment success.

## Figures and Tables

**Table 1:** Clinical features of LETM reported previously within 3 years.

Authors	Year	Age	Gender	The extent of spinal cord lesion on MRI findings	Initial symptoms	Pathogenesis	Prognosis
Graham et al. [14]	2013	25	Male	T3-L2	Spastic paraparesis	Neuro-Behçet's disease	Improved

Huang et al. [13]	2013	39	Male	C3-conus medullaris	Motor and sensory disturbance in lower limbs	SLE	Not improved

Salazar et al. [12]	2013	46	Male	Entire spinal cord	Leg weakness, ataxia, and paresthesia	NMO/HIV	Improved

Coulter et al. [11]	2012	18	Male	T3-conus medullaris	Spastic paraplegia and pyramidal weakness	Neuro-Behçet's disease	Improved

White et al. [10]	2012	18	Male	T3-conus medullaris	Numbness and flaccid paralysis in lower limbs	SLE	Improved

Stanifer et al. [9]	2012	50	Female	C4-T4	Motor and sensory disturbance in all four limbs	Sjögren's syndrome	Improved

Franciotta et al. [8]	2011	62	Female	C6-T11	Right leg weakness and numbness in lower limbs	NMO	Improved

Habek et al. [7]	2011	43	Female	Medulla oblongata to C7	Spastic tetraparesis	NMO spectrum disorder	Improved

Itami et al. [6]	2011	73	Female	Entire spinal cord	Gait disturbance	HTLV-1-associated myelopathy	Improved

Kumar et al. [5]	2011	82	Female	T3-T11	Lower limbs weakness and numbness	Intravascular lymphoma	Died

Nightingale et al. [4]	2011	78	Male	C5-T10	Bilateral leg weakness	NMO	Not improved
		31	Female	C5-T4	Bilateral leg weakness and numbness	NMO spectrum disorder	Improved

Ohnaka et al. [3]	2010	34	Male	From T3 vertebral body to conus medullaris	Paraplegia in lower limbs	Lung cancer	Symptoms remained

Akkad et al. [2]	2010	27	Female	From the top of cervical vertebrae to thoracic cord	Weakness and paresthesias in lower limbs	Vaccination against influenza vaccine	Improved
